# MMP2-A2M interaction increases ECM accumulation in aged rat kidney and its modulation by calorie restriction

**DOI:** 10.18632/oncotarget.23652

**Published:** 2017-12-24

**Authors:** Kyung Mok Kim, Ki Wung Chung, Hyeong Oh Jeong, Bonggi Lee, Dae Hyun Kim, June Whoun Park, Seong Min Kim, Byung Pal Yu, Hae Young Chung

**Affiliations:** ^1^ Molecular Inflammation Research Center for Aging Intervention (MRCA), College of Pharmacy, Pusan National University, Busan, Republic of Korea; ^2^ Korean Medicine (KM)-Application Center, Korea Institute of Oriental Medicine (KIOM), Daegu, Republic of Korea; ^3^ Department of Physiology, The University of Texas Health Science Center at San Antonio, San Antonio, TX, USA

**Keywords:** Aging, renal fibrosis, MMP2, A2M, extracellular matrix, Gerotarget

## Abstract

Age-associated renal fibrosis is related with renal function decline during aging. Imbalance between accumulation and degradation of extracellular matrix is key feature of fibrosis. In this study, RNA-sequencing (RNA-Seq) results based on next-generation sequencing (NGS) data were analyzed to identify key proteins that change during aging and calorie restriction (CR). Among the changed genes, A2M and MMP2, which are known to interact, exhibited the highest between centrality (BC) and degree values when analyzed by protein–protein interaction (PPI). Both mRNA and protein levels of MMP2 and A2M were increased during aging. Furthermore, the interaction between MMP2 and A2M was verified by immunoprecipitation and immunohistochemistry. MMP2 activity was further measured under the presence or absence of A2M-MMP2 interaction. MMP2 activity, which was increased under the absence of A2M-MMP2 interaction, was significantly decreased under the presence of interactions in aged kidney. We further hypothesized that the interaction between A2M-MMP2 played a role in the inactivation of MMP2 leading to accumulation of ECM including collagen type I and IV. Aged kidney showed highly accumulated MMP2 substrate proteins despite of increased MMP2 protein expression and CR blunted these accumulation. Additional *in vivo* analysis revealed that the signal transducer and activator of transcription (STAT) 3 transcriptional factor was significantly increased thus increasing A2M expression during aging. STAT3 activating cytokines were also highly increased in aged kidney. In conclusion, the results of the present study indicate that A2M-MMP2 interaction has a role in age-associated renal ECM accumulation and in the suppression such fibrosis by CR.

## INTRODUCTION

The main characteristic of biological aging is defined as structural and functional degeneration due to the passage of time, and that degeneration tends to increase susceptibility to various diseases including diabetes, cardiovascular diseases, dementia, and other chronic diseases [1]. Currently, the study of aging is rapidly advanced, notably due to the recent elucidation that the rate of aging is regulated, at least to some extent, by genetic and biochemical processes conserved in evolution [2]. Among the regulation that can extend the lifespan, calorie restriction (CR) has been studied by many gerontology researchers and it is accepted as the golden standard for lifespan extension and health improvement [3]. It has been suggested that CR can retard aging by modulating defective energy metabolism and oxidative stress. Although it is evident that CR can result in decline in the accumulation of age-associated damages, the anti-aging ability of CR still remains unclear.

Among the changed organs during aging, aging produces the most dramatic changes in kidney [4]. The major features of renal aging are glomerulosclerosis, tubular atrophy and interstitial fibrosis [5]. These pathological changes ameliorate renal function during aging. Interestingly, recent study suggested that ECM accumulation is an early indicator of renal aging that can connect renal structure changes to functional decline [6]. It has been reported that CR greatly reduces several risk factors related to kidney aging [7]. For example, kidney aging indicators such as glomerular enlargement, podocyte hypertrophy, and mitochondrial abnormality have been delayed or even reversed by CR [7, 8]. Similarly, other researchers have shown that CR attenuates most of the analyzed age-related markers in kidney, such as glomerular basement membrane (GBM) thickness, mitochondrial mass in complex proximal tubules and autophagic markers in aged kidney [9]. Furthermore, CR has been shown to decrease age-associated renal fibrosis and chronic kidney diseases [10]. However, biological processes how CR can retard excessive renal fibrosis needs to be demonstrated.

MMPs are a large family of extracellular zinc-dependent endopeptidases comprising 23 members that are able to degrade ECM components [11]. MMPs regulate a broad spectrum of physiological and pathological processes including cell proliferation, angiogenesis, metastasis, immunity, wound healing, and tissue remodeling [12]. Because the MMPs robustly participate in degradation of ECM components under various physiological and pathological conditions, the importance of MMPs has been emphasized in repairing tissue damage during wound healing and ECM remodeling [12]. In other words, the rate of tissue remodeling during tissue damage is primarily modulated through the activity of MMPs. Therefore, it is well established that MMP family is a major regulator of renal ECM homeostasis [13]. MMP2, called gelatinase A, is a type IV collagenase that can cleave type I and type IV collagens which are major components of ECM. In addition, MMP2 has many roles in physiological processes including embryonic development and reproduction as well as in pathogenic processes such as arthritis and metastasis. The regulation of MMP2 activity has been implicated in several fibrosis model [11]. Dys-regulated MMP2 levels or activities has been strongly associated with excessive ECM accumulation leading to fibrotic diseases.

As a major proteinase inhibitor, A2M binds most proteinases with broad specificity [14]. It is a large glycoprotein consisting of four identical 185 kDa subunits. Two of those subunits are disulfide-bonded to each other and half molecules are attached non-covalently. A2M interacts with various kind of proteinases including plasmin, kallikrein, thrombin, and gelatinases (MMP2 and MMP-9) [14, 15]. A2M is also known to act as carrier protein because it also binds to numerous growth factors and cytokines. Generally, A2M is mainly produced by liver as an acute phase protein during the stress states. A2M is further secreted into blood and extracellular spaces where it fulfills its function. Furthermore, it is also locally produced by macrophages, fibroblasts, and epithelial cells. Interestingly, increased A2M levels are detected in some nephrotic syndromes indicating the important role of A2M in kidney [16].

Age-associated renal fibrosis has been recognized as a common pathway leading to renal dysfunction during aging. When the loss of renal function is progressed seriously, it leads to end stage renal diseases (ESRD), which is irreversible, eventually. ECM accumulation is a major characteristic of renal fibrosis. Therefore, it is important to find crucial factors that can maintain the balance between synthesis and degradation of ECM. Although the roles of MMP2 in the regulation of ECM degradation have been extensively studied, its role in aging has not been reported. In addition, an interaction between A2M and MMP2 in aged kidney has not been previously reported. The purpose of this study is to investigate the role of the A2M-MMP2 interaction in age-associated renal fibrosis.

## RESULTS

### Importance of A2M-MMP2 interaction during aging and CR identified by RNA sequencing and protein-protein interaction analysis

**Figure 1:**
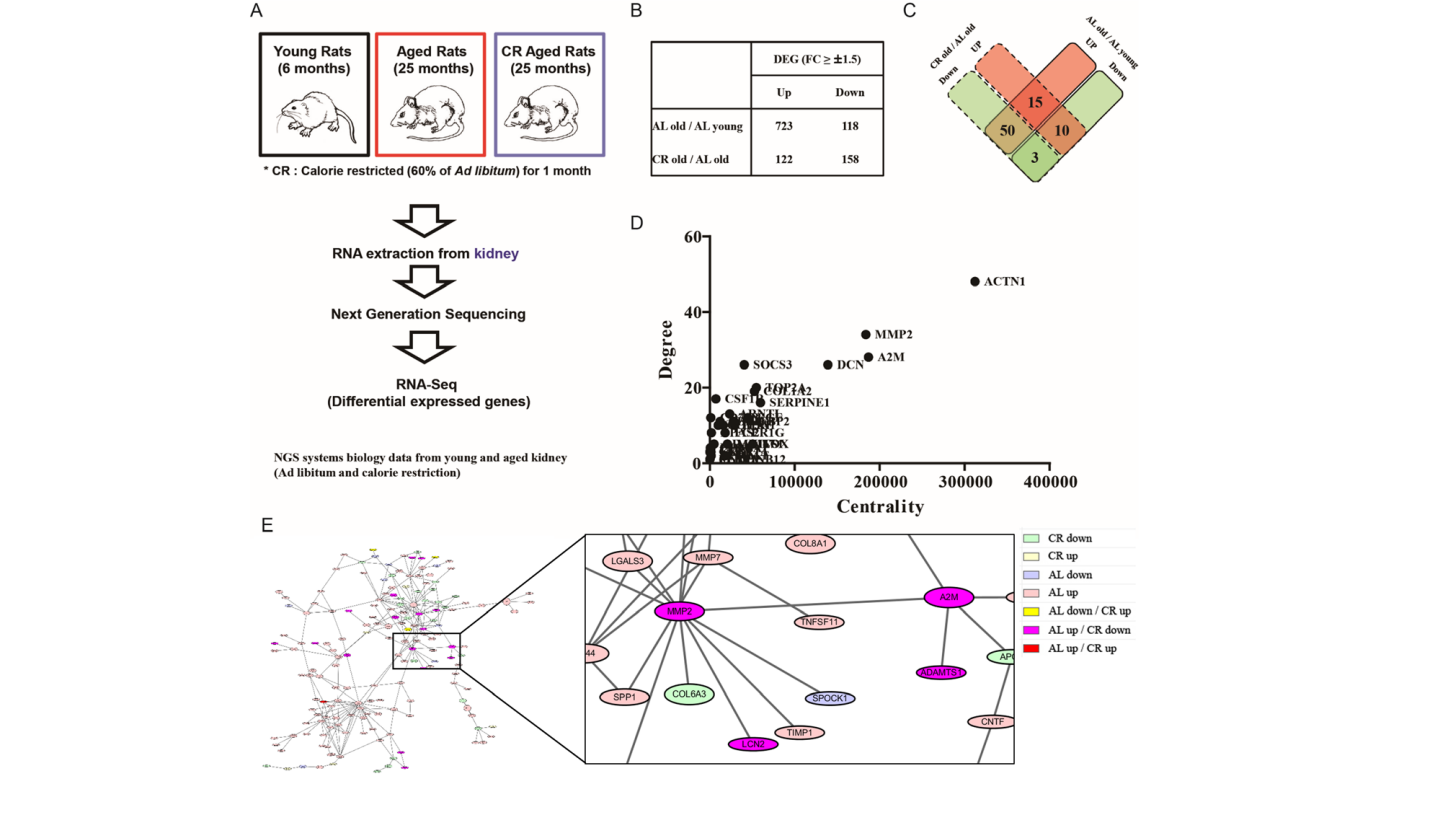
Importance of MMP2 and A2M interaction during aging identified by RNA-Seq based on NGS data (**A**) Brief scheme of NGS analysis from young, aged, and CR rat kidney. RNA-Seq technology based on Illumina Hiseq-2000 was utilized to investigate molecular changes during aging and CR. (**B**) Result of genes changed during aging and CR analyzed by identifying DEG. (**C**) Result of commonly changed genes during aging and CR. (**D**) Degree and centrality of commonly changed genes. The degree and centrality of A2M and MMP2 are highest among the commonly changed genes. (**E**) Result of PPI analysis among the commonly changed genes during aging and CR. Result shows the direct interaction of A2M-MMP2. The colors indicate the changes of gene expression by aging and CR. Gene expressions of A2M and MMP2 are both increased by aging and decreased by CR.

To evaluate gene changes during aging and CR, NGS data were analyzed by applying RNA-Seq methods. Briefly, RNA was extracted from kidney of 6-month-old (young), 25-month-old (aged) rats fed a regular diet and 25-month-old (aged) CR (60% CR for one month) rats. Subsequently, whole genome sequencing was performed by performing NGS. Using the obtained data, DEG was analyzed via RNA-Seq to identify genes that changed expression during aging and CR (Figure 1A). Compared with young rats, the number of genes up-regulated in aged rats was 723 and the number of down-regulated genes was 118. Moreover, compared to aged rats fed a regular diet, the number of up-regulated genes in aged CR rats was 122 and there were 158 down-regulated genes (Figure 1B). Among these various gene changes, we focused on genes that changed both aged and aged CR rats. The number of genes that were up-regulated in aged rats and down-regulated in CR rats was 50 (Figure 1C). To elucidate the most important gene changes, PPI was analyzed among all changed genes. PPI analysis can predict interactions between two proteins based on previously reported data. Once, PPI results were obtained, BC was calculated to identify the proteins that were most related to aging and CR. (Figure 1D). BC includes both degree and centrality, with degree results showing the number of interactions between proteins. Moreover, proteins which have a bridging role between clusters can have high centrality. Therefore, proteins possessing a high BC can play vital roles. The PPI analysis of all changed genes that were up-regulated with aging and down-regulated with CR showed that MMP2 had both high degree and high centrality (Figure 1D). Furthermore, A2M, which directly interacted with MMP2, also showed high degree and high centrality (Figure 1D). Interestingly, both genes were up-regulated by aging and down-regulated by CR and shown to be directly associated (Figure 1E). These data indicate the importance of MMP2, A2M, and A2M-MMP2 interaction during aging and CR.

### Validation of MMP2 and A2M expression during aging and CR

**Figure 2:**
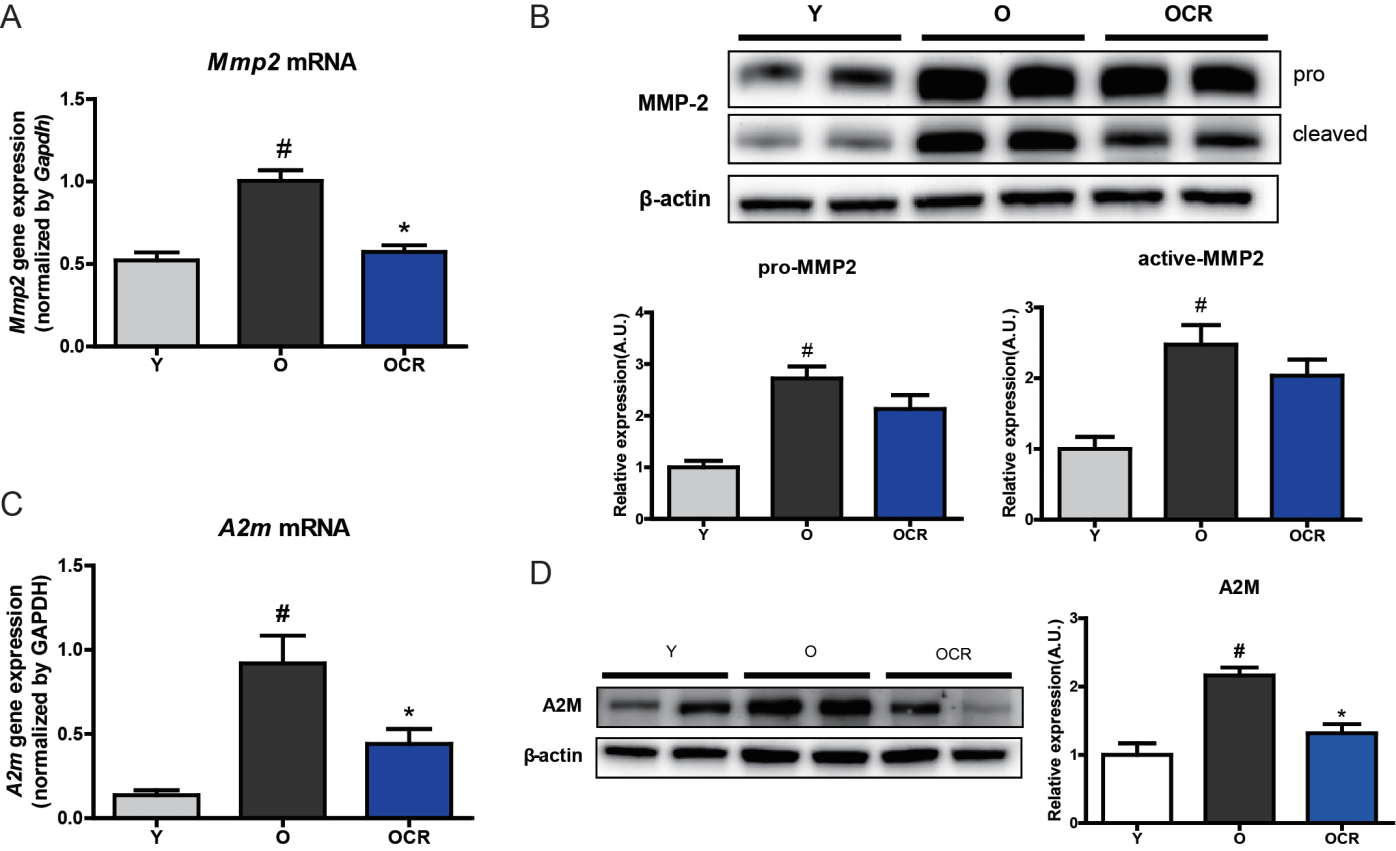
Alteration of MMP2 and A2M expression during aging and CR (**A**) Gene expression on MMP2 was detected by qPCR. The mRNA levels were normalized to the *Gapdh* level. #P < 0.05 *vs* young. *P < 0.05 *vs* old. (**B**) Western blotting was conducted to detect protein expression of MMP2. β-actin was used as loading control. The blots were quantified by densitometry (n = 8). Data are expressed as the mean ± SEM. #P < 0.05 *vs* young. (**C**) Gene expression of *A2m* is detected by qPCR. mRNA levels were normalized to the *Gapdh* level. #P < 0.05 *vs* young. *P < 0.05 *vs* old. (**D**) Protein level of A2M is identified by Western blotting. The blots were quantified by densitometry (n = 8). Data are expressed as the mean ± SEM. #P < 0.05 *vs* young. *P < 0.05 *vs* old.

To confirm the previously observed NGS analysis results, qPCR was performed to observe *MMP2* gene expression. The mRNA expression of *MMP2* significantly increased within aging and decreased with CR, supporting the results of the NGS analysis (Figure 2A). To check whether increased MMP2 mRNA expression is associated with protein expression, Western blotting was further performed. Protein expression of pro- and active- forms of MMP2 was also significantly increased in aged rat kidney (Figure 2B). However, although CR decreased the pro- and active- forms of MMP2 in aged rats, the difference was not statistically significant (Figure 2B). We further checked, A2M, which was shown by the PPI results to be directly connected with MMP2, levels in our model. *A2m* gene level was analyzed by qPCR. *A2m* gene levels were increased by aging, whereas CR significantly decreased *A2m* gene expression (Figure 2C). In addition, protein levels were also significantly increased by aging. Interestingly, the A2M protein level in aged CR rat kidney was almost the same as that in young rats (Figure 2D). These results indicate that both MMP2 and A2M mRNA expression and protein levels were up-regulated by aging, whereas CR only affected A2M protein expression induced by aging.

### Effects of A2M-MMP2 interaction on MMP2 activity in kidney

**Figure 3:**
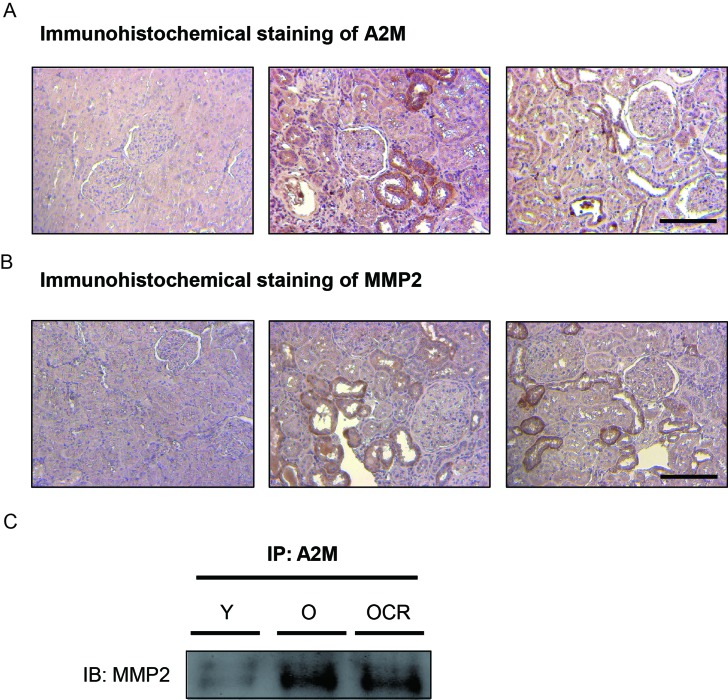
Effects of aging and CR on A2M-MMP2 interaction (**A**) The expression of A2M is visualized by immunohistochemical methods. A2M was dyed brown in color in young, aged, and CR kidney tissue sections via immunohistochemistry. Slides were counter-stained with haematoxylin in blue. Scale bar = 50 μm. (**B**) The expression of MMP2 is visualized by immunohistochemical methods. A2M was dyed brown in color in young, aged, and CR kidney tissue sections via immunohistochemistry. Slides were counter-stained with haematoxylin in blue. Scale bar = 50 μm. (**C**) The interaction between A2M and MMP2 was detected by immunoprecipitation.

We further focused on the interaction between A2M and MMP2 during aging and CR. First, immunohistochemical (IHC) staining of A2M and MMP2 were performed. IHC staining of A2M showed increased expression of A2M in the interstitial or damaged tubule segment of kidney during aging compared to young (Figure 3A). CR significantly reduced A2M expression in parallel with protein expression data (Figure 3A). Furthermore, IHC results of MMP2 also indicated increased expression in the interstitial or damaged epithelial region during aging (Figure 3B). Unlike A2M expression, MMP2 expression were not affected by CR (Figure 3B). We further examined direct interaction between A2M-MMP2 by immunoprecipitation. The results showed that aging increased the A2M-MMP2 interaction, whereas CR significantly decreased that interaction (Figure 3C). These results demonstrated that aging induces increased interaction between MMP2 and A2M, and CR decreases the interaction between MMP2 and A2M.

**Figure 4:**
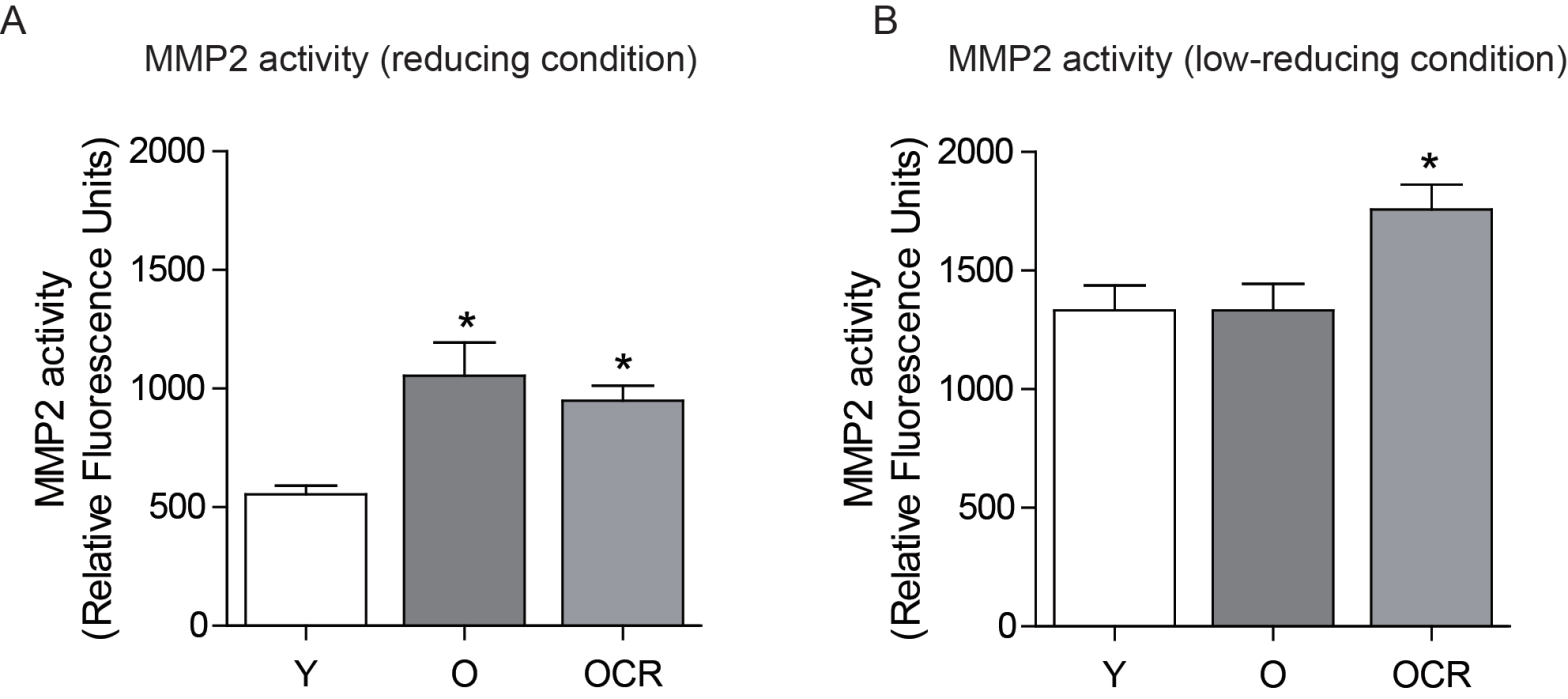
Effects of aging and CR on MMP2 activity (**A**) MMP2 activity was measured under reducing condition (5% SDS) in young, aged, and CR kidneys using fluorescent dye. (**B**) MMP2 activity was measured under low reducing condition (0.1% SDS) in young, aged, and CR kidneys using fluorescent dye.

Next, we measured MMP2 activity by using fluorogenic substrates of MMP2. Under the highly reducing condition with the inhibition of interactions, MMP2 activity was significantly higher in aged kidney compared to young kidney (Figure 4A). The activity was not decreased in CR groups (Figure 4A). Under the low-reducing condition, over-all activities of MMP2 were increased compared to reducing condition (Figure 4B). However, the activity of young and aged kidney MMP2 was almost same, while the activity of MMP2 in CR was still significantly higher compared to young or aged kidney (Figure 4B). These results indicated that MMP2 activity in aged kidney was not different from young kidney under the low-reducing condition, implying the interactions between MMP2 and other proteins are important in MMP2 activity regulation.

### Effects of aging and CR on extracellular matrix accumulation and fibrosis

**Figure 5:**
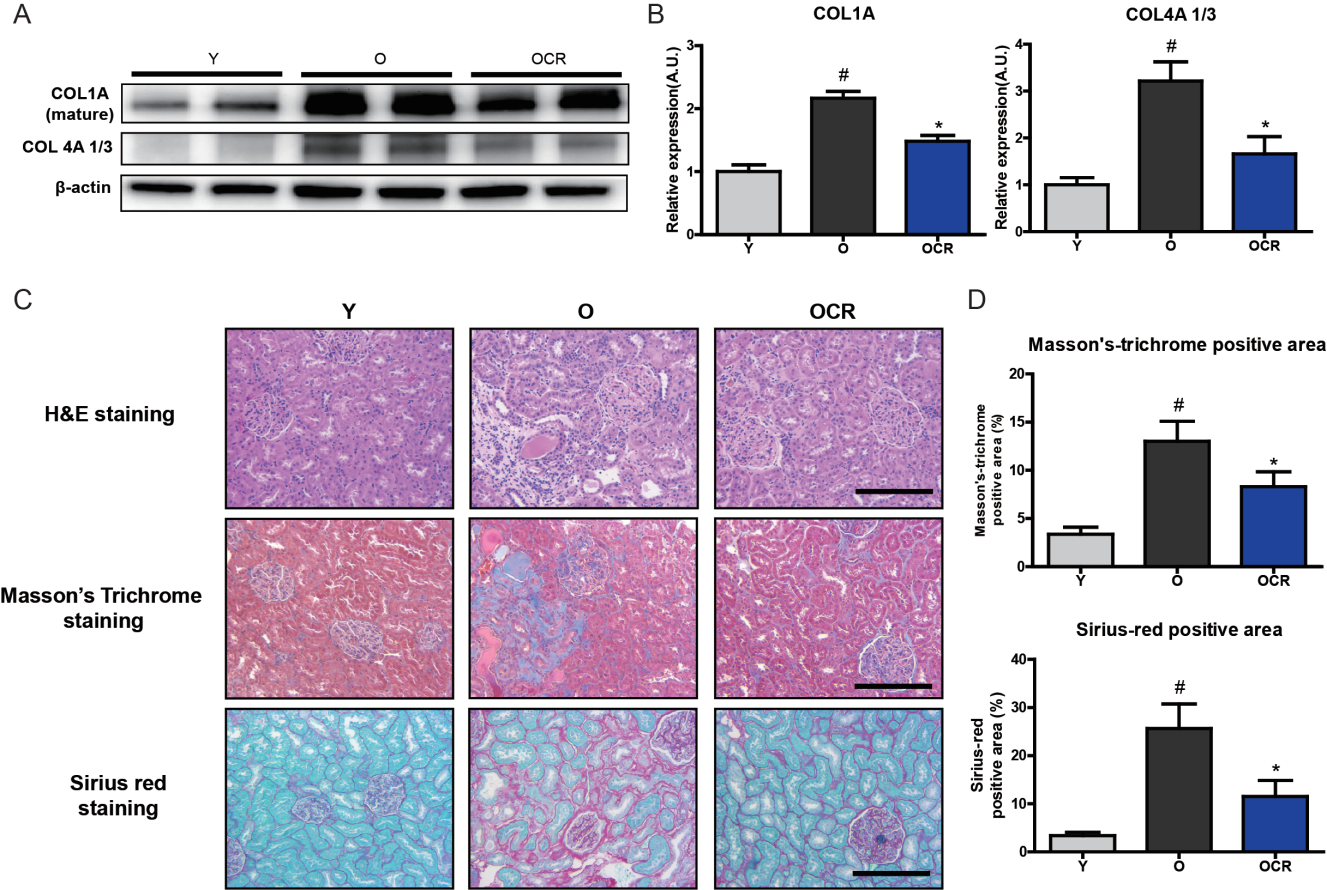
Changes of MMP2 substrate extracellular matrix proteins and fibrosis during aging and CR (**A**) Accumulation of type I and IV collagens during aging in kidney is detected by Western blotting. β-actin was used as loading control. (**B**) The blots in figure 5A were quantified by densitometry (n = 8). #P < 0.05 *vs* young. *P < 0.05 *vs* old. (**C**) ECM accumulation in young, aged, and CR kidney is visualized by staining with Masson’s Trichrome (MT) and Sirius red staining. Representative pictures of staining results are presented. MT staining and Sirius red staining positive areas were quantified. Both results show that aging increased ECM accumulation and CR attenuates these accumulation. Scale bar = 50 μm. (**D**) Quantification of Sirius red and MT stained fibrosis in kidneys. #P < 0.05 *vs* young. *P < 0.05 *vs* old.

Next, we checked the substrates of MMP2, collagen I and collagen IV, which are known to be accumulated during aging. Both collagens, two of the most widely reported substrates of MMP2, were significantly increased by aging, despite the increase in MMP2 protein level with aging (Figure 5A, 5B). Interestingly, although MMP2 protein expression was not significantly decreased by CR, both type I and IV collagens accumulations were reduced in CR rat kidney (Figure 5A, 5B). The extent of ECM accumulation during aging and CR was further analyzed by staining with Masson’s trichrome (MT) and Sirius red (SR) staining. The results showed that aging considerably increased ECM accumulation in renal tubule-interstitial space as well as in the glomerular region, whereas CR reduced ECM accumulation (Figure 5C). The quantification of MT and SR staining positive regions also indicated increase of ECM during aging and reduction by CR (Figure 5D). Collectively, these results demonstrated that substrates of MMP2 were increased during aging regardless of increased MMP2 expression, and CR significantly decreased the accumulation of MMP2 substrates during aging.

### Analysis of STAT3 activation and STAT3 inducing factors during aging and CR

**Figure 6:**
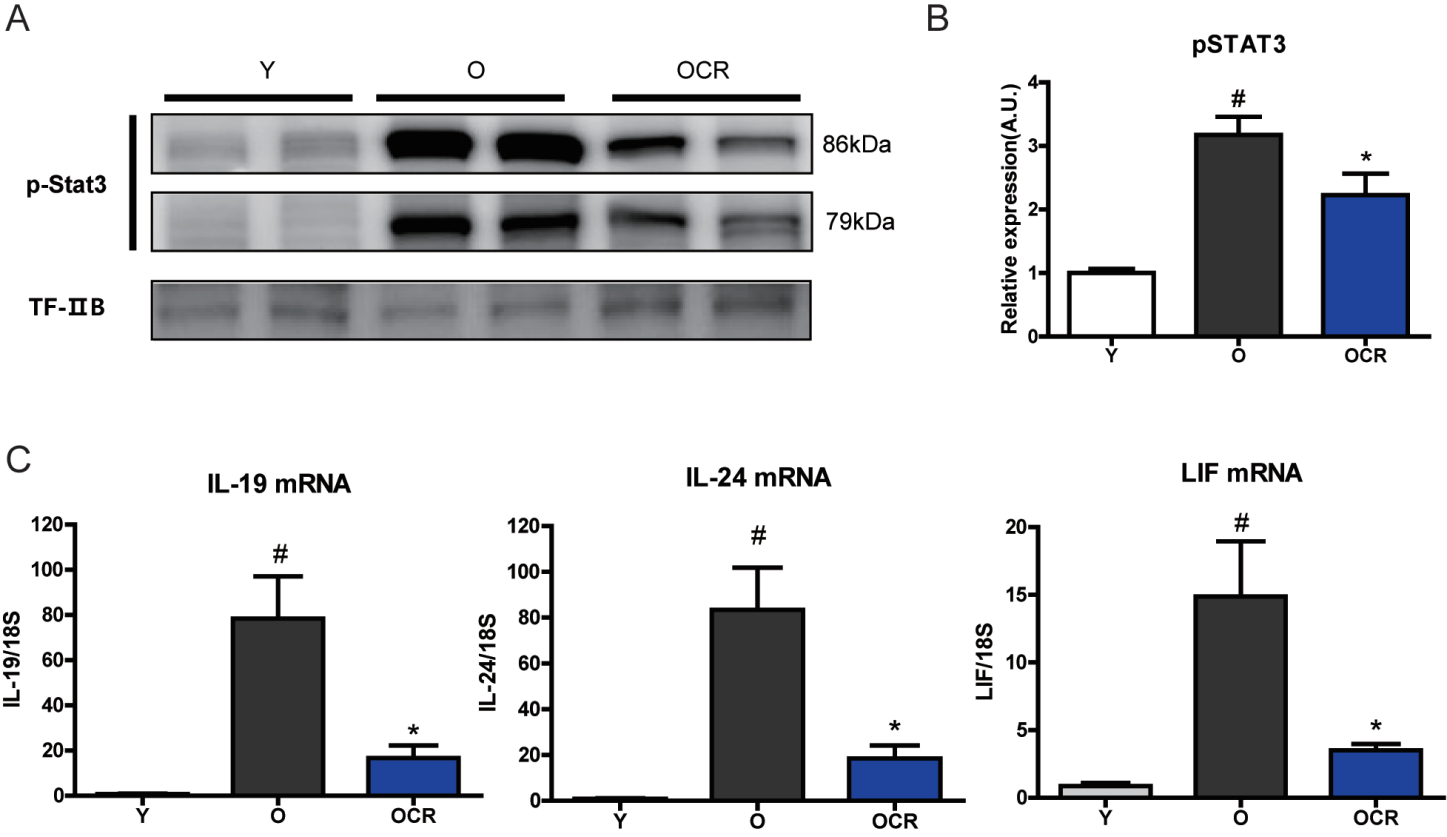
Effects of aging and CR on STAT3 activation and STAT3 inducing cytokines (**A**) Phosphorylated (activation form) STAT3 is detected by Western blotting in the nuclear fraction of kidney. The blots were quantified by densitometry (n = 8). TFIIB was used as the loading control. (**B**) The blots in figure 6A were quantified by densitometry (n = 8). #P < 0.05 *vs* young. *P < 0.05 *vs* old. (**C**) Gene expressions of IL-19, IL-24, and LIF were detected by qPCR. mRNA levels were normalized to the *18S* level. #P < 0.05 *vs* young. *P < 0.05 *vs* old.

It has been reported that the STAT3 transcriptional factor has a critical role in *A2m* expression [17]. STAT3 is activated by various ligands binding to receptors induce JAK activation. Activated JAK signaling directly phosphorylates STAT3 and phosphorylated STAT3 plays a role in the transcription of various genes including *A2m*. The level of phosphorylated STAT3 was significantly increased in aged kidney, whereas the phosphorylation level of STAT3 in CR kidney was decreased (Figure 6A, 6B). Because STAT3 activation was dramatically increased in aged kidney, factors that can induce STAT3 phosphorylation were screened. Various cytokines, growth factors, and other hormones can activate the STAT3 signaling pathway. Among the various factors, IL-19 and IL-24, which are members of the IL-20 cytokine family, were markedly increased in aged kidney (Figure 6C). Compared to mRNA levels in young rat kidney, the mRNA expressions of *IL-19* and *IL-24* increased by 91.4- and 83.3-fold, respectively, in aged rat kidney. Moreover, CR significantly attenuated the age-induced *IL-19* and *IL-24* increases (Figure 6C). Another STAT3 activation factor, LIF, was also significantly increased by aging and reduced by CR (Figure 6C). Other STAT3 inducing factors showed marginal effects during aging and CR (Supplementary Figure 1). These results implicate that an increase in A2M during aging might be the result high level of activated STAT3 during aging.

## DISCUSSION

In the present study, we investigated into the role of MMP2-A2M interaction on ECM accumulation during aging. The importance of MMP2-A2M interactions were derived from analysis of RNA-sequencing followed by PPI analysis in young, aged, and CR kidneys. Further biochemical and histological assays revealed increase of MMP2 and A2M expression as well as their interaction during aging. Critically, the activity of MMP2 was affected by different reducing conditions in aged kidney, implicating the importance of interactions in the activity modulation. We further checked the expression of MMP2’s substrate including type I and type IV collagens. Regardless of increased MMP2 expression during aging, the expression of substrate were also significantly increased during aging. We assumed that the increased interaction between A2M-MMP2 during aging decreased MMP2’s activity leading to excessive accumulation of ECM. Additional analysis revealed that A2M-inducing STAT3 transcriptional factor and STAT3 inducing factors were significantly increased by aging. CR significantly reduced A2M-MMP2 interactions and increased MMP2 activity with decreased ECM accumulation induced by aging. Collectively, our data suggest the involvement of A2M-MMP2 interaction in the regulation of ECM accumulation during aging and anti-aging CR reduces ECM accumulation by decreasing the interactions (Supplementary Figure 2).

Aging is commonly defined as progressive functional decline that leads most living organisms through an irreversible biological process with the passage of time [2]. A universal view of the several hallmarks of the aging process including genomic instability, telomere attrition, epigenetic alterations, loss of proteostasis, deregulated nutrient sensing, mitochondrial dysfunction, cellular senescence, and stem cell exhaustion have been studied by researchers around the world [2]. However, there are still numerous challenges in relation to fully describing this complex biological process [18]. It has been recently suggested that aging is associated with progressive ECM accumulation which involves loss of function in several tissues [19-22]. Because kidney is one of the most vulnerable tissues which can be influenced by aging and age-related physiological changes, it also shows time-dependent accumulation of ECM in the glomerulus and interstitial parts followed by fibrosis [6, 23]. In our study, ECM accumulation was identified in aged rat kidney by analyzing Western blotting and immunohistochemical results. It is also well established that age-related structural changes in kidney occur earlier than previously expected, thus indicating the importance of renal fibrosis to functional changes in kidney [24]. Although it is evident that aging increases ECM accumulation in kidney, the exact mechanisms are not fully addressed.

CR has been studied by many gerontology researchers and it is accepted as the golden standard for lifespan extension and health improvement. We also found that CR significantly reduces ECM accumulation during aging. CR reduced type I and IV collagen accumulation induced by aging. Taken together, other’s reports and our results show that CR effectively reverses or protects against kidney function decline and fibrosis during aging through its modulating effects on renal biology.

There are conflicting reports on the role of MMPs during fibrogenesis. At first sight, it seems that MMPs are anti-fibrotic because of its reported role in the degradation of matrix proteins. However, there are also pro-fibrotic roles due to induction of tubular cell EMT [11]. In a previous study, for example, there was an early increase in MMP2 expression and activity in a unilateral ureteral obstruction model. Those results indicate an increase in ECM turnover following injury, but reduced degradation favoring the development of tubulointerstitial fibrosis at a later time [25]. Results of another study of an MMP2 transgenic mouse model suggested a pro-fibrotic role of MMP2 [26]. These mice mimic human CKD, including tubular atrophy, glomerulosclerosis, and tubulointerstitial fibrosis, thus suggesting a role of MMP2 in CKD. However, in a diabetic nephropathy model, MMP2 knockout mice showed increased collagen deposition and fibroblast activation suggesting an anti-diabetic and anti-fibrotic role of MMP2 [27]. These divergent research results have made clarifying, the role of MMP2 in ECM accumulation during fibrogenesis an interesting topic [28]. Our result also indicates the importance of MMP2 in age-associated renal fibrosis. Although MMP2 expression was significantly increased during aging, the activity of MMP2 was not comparable with its expression. Thus, we hypothesized that there may be some other mechanisms that controls MMP2 activity during aging.

The activity of MMP2 can be regulated through several mechanisms. First, the expression of MMPs is important, and when needed expression of most MMPs is regulated at the transcription level. In this study, *MMP2* gene expression was up-regulated in aged rat kidney. In addition, MMP2 protein expression was also increased. Although active MMP2 proteins were up-regulated during aging, the substrates of MMP2 including type I and IV collagens also accumulated leading to severe fibrosis. Therefore, it was assumed that there might be other regulators other than the one that only controls MMP2 transcription. Such second mechanisms include various controls over enzyme activity including zymogen activation and compartmentalization of active proteinase processes. These mechanisms include the suppression of MMPs by anti-proteinases [11].

In this study, the interaction between MMP2 and A2M was focused upon due to results of the PPI analysis of aged rat kidney. A2M, one of the anti-proteinases known to bind and inactivate MMP2, was up-regulated during aging. The interaction between MMP2 and A2M was detected by two methods in our experiments. Increased MMP2 and A2M regions were quite similar when detected by immunohistochemistry. Furthermore, more directly, the increased interaction between MMP2 and A2M was verified by immunoprecipitation. Because MMP2 activity was significantly affected by reducing state that regulates interactions only in aged kidney, it is plausible that A2M-MMP2 interactions during aging may resulted in decrease of MMP2 activity during aging.

The synthesis of A2M is primarily regulated at the transcriptional level, and activation of the STAT3 transcriptional factor increases *A2m* gene expression [29]. It has also been shown that the *A2m* gene is activated by IL-6 through the action of STAT3 [30]. Transfection experiments have identified two binding sites for STAT3 on *A2m* [30]. The STAT proteins are latent in the cytoplasm and, following IL-6 induction, activated STAT3 promotes an increase in *A2m* gene transcription [29]. In our experiments, activation of STAT3 detected by its phosphorylation was dramatically increased during aging indicating the possible role for A2M expression. In addition, STAT3 activating cytokines, especially IL-19, IL-24, and LIF were also dramatically increased. Further studies will be needed to elucidate the exact mechanisms and the cell types expressing A2M in aged kidneys.

## MATERIALS AND METHODS

### Animals

To investigate the effects of an interaction between A2M and MMP2 on age-associated renal ECM accumulation, male SD rats aged 6 (young) and 25 (aged) months (Samtako, Osong, Korea) were used. Rats were divided into three groups. The standard diet (AL) fed group (young and aged) had free access to both food and water. The CR rat group (aged) was fed 60% of the food intake of their AL-fed littermates for 1 month. Eight rats were included in each group. Rats were maintained under a 12 h light/dark cycle at 23 ± 1°C and 50 ± 5% relative humidity under specific pathogen-free conditions. Serum was collected for biochemical analysis. Kidneys were extracted and either frozen immediately in liquid nitrogen for RNA-sequencing analysis, quantitative polymerase chain reaction (qPCR), Western blotting, and other biochemical analyses, or fixed in neutral-buffered formalin for histochemical examination. The animal protocols for ethical procedures and scientific care that were used in this study were reviewed by the Pusan National University-Institutional Animal Care and Use Committee (PNU-IACUC).

### RNA-sequencing analysis from NGS data

Total RNA was extracted from each sample by using the miRNeasy Mini Kit (Qiagen, Hilden, Germany). An RNA sequencing library was generated by using the TruSeq RNA sample preparation Kit according to user’s instruction manual (Illumina, San Diego, CA, USA). Briefly, mRNA was isolated from total RNA using Oligo(dT) beads and was chemically fragmented. After double-strand cDNA synthesis of the fragmented mRNA, end-repair, adenylation of the 3′-end, and sequencing adapter ligation were performed, followed by DNA purification with magnetic beads and PCR amplification. Finally, the amplified library was purified, quantified, and then applied for template preparation. The HiSeq2000 platform was utilized to generate 99-bp paired-end sequencing reads (Illumina).

### Tissue preparation and protein extraction

All solutions, tubes, and centrifuges were maintained at 0-4°C. Tissue samples (100 mg to 200 mg) of frozen kidney were homogenized with 1ml of homogenate buffer containing 100 mM Tris, 20 mM β-glycerophosphate, 20 mM NaF, 2 mM sodium orthovanadate, 1 mM EDTA, 0.01 mM dithiothreitol (DTT), 0.5 mM phenylmethylsulfonyl fluoride (PMSF), 1X Protease inhibitor cocktail solution (GenDPOT, Barker, TX, USA) by using a tissue homogenizer for 30 sec and then kept on ice for 20 min. Next, 125 μl of 10% Nonidet P-40 (NP-40) solution was added, mixed for 15 sec, and centrifuged at 12,000 *rpm* at 4°C for 5 min. Supernatants were used as cytosolic fractions and the pellets were washed once with 400 μl homogenate buffer with 50 μl of 10% NP-40, centrifuged, suspended in 100 μl of a buffer containing 50 mM KCl, 300 mM NaCl, 0.1 mM EDTA, 10% (v/v) glycerol, 0.01 mM DTT, 20 mM β-glycerophosphate, 20 mM NaF, 2 mM sodium orthovanadate, 1 mM EDTA, 0.5 mM PMSF, 1X protease inhibitor cocktail, kept on ice for 30 min, and centrifuged at 12,000 *rpm* at 4°C for 10 min. The resultant supernatants were used as nuclear fractions. Protein concentration was measured by the bicinchoninic acid (BCA) assay method and using bovine serum albumin (BSA) as the standard.

### Western blotting

Western blot assays were performed as described previously with minor modification (Gershoni & Palade 1983). Cytosolic or nuclear proteins (20∼100 μg of protein) were boiled for 5 min in gel-loading buffer (60 mM Tris–HCl, pH 6.8, 2 % SDS, 25% glycerol, 10 % 2-mercaptoethanol, and 0.1 % bromophenol blue) at a volume ratio of 1:1. Samples containing the same amounts of proteins were then separated by sodium dodecyl sulfate–polyacrylamide gel electrophoresis in 6 % ∼ 15 % acrylamide gels and transferred by using a Bio-Rad western system (Bio-Rad, Hercules, CA, USA) to PVDF membranes, which were immediately placed in blocking buffer (5% non-fat milk) containing 10 mM Tris (pH 7.5), 100 mM NaCl, and 0.1% Tween 20. Membranes were then washed in TBS-Tween buffer for 30 min, incubated with specific primary antibodies (dilution 1:500 to 1:2,000, Supplementary Table 1) at 4 °C overnight, washed for 3x10-min in TBS-Tween buffer, and incubated with horseradish peroxidase-conjugated anti-mouse antibody (Santa Cruz, 1:10,000), anti-rabbit antibody (Santa Cruz, 1:10,000), or anti-goat antibody (Santa Cruz, 1:10,000) at 25 °C for 1 h. The resulting immunoblots were visualized by using Western Bright Peroxide solution (Advansta, CA, USA) and Davinch-chemi CAS-400 (Davinch-K, Seoul, Korea), according to the manufacturer’s instructions.

### Isolation of total RNA and qPCR

Total RNA was isolated by the method reported in a previous study [31]. Briefly, tissue samples were homogenized in the presence of RiboEX™ (GeneAll, Seoul, Korea) (1 ml per 50 mg tissue) by applying a few strokes in a tissue homogenizer. An aliquot of 0.2 ml chloroform per 1 ml homogenate was added, and the samples shaken vigorously for 15 sec. The aqueous phase was transferred to a fresh tube, to which an equal volume of isopropanol was added, and the samples were kept at 4°C for 15 min, Samples were again centrifuged at 8,000 *rpm* at 4°C for 15 min. The supernatant was removed and the RNA pellet was washed once with 75 % ethanol by vortexing and subsequent centrifugation at 8,000 *rpm* at 4 °C for 8 min. The pellet was dried for 10-15 min. The RNA pellet was dissolved in DEPC-treated water. RNase-free DNase-treated total RNA (1.5 μg) was reverse-transcribed with a 2X Hyperscript RT master mix (with Random Hexamer) (GeneAll, Seoul, Korea). The cDNA was stored at -20°C until use. The synthesized cDNA was used as a template for real-time PCR, which was performed by using SYBR green real-time master mix (Geneall, Seoul, Korea). Primer sequences are shown in Supplementary Table 2. Real-time PCR and data analyses were performed using the CFX Connect System (Bio-Rad Laboratories, Hercules, CA, USA).

### Immunoprecipitation

Approximately 500 μg of kidney protein was immunoprecipitated in a buffer containing 40 mM Tris-HCl (pH 7.6), 120 mM NaCl, 20 mM glycerophosphate, 20 mM NaF, 2 mM sodium orthovanadate, 5 mM EDTA, 1 mM PMSF, 0.1% NP40, 1X Protein inhibitor cocktail. Aliquots of kidney homogenates were centrifuged at 12,000 *rpm* at 4 °C for 15 min, incubated overnight at 4 °C with A2M antibodies, and then incubated overnight at 4 °C with 50 % protein A-agarose slurry. After washing the immunoprecipitates three times with immunoprecipitation buffer, the immunoprecipitated proteins were analyzed by SDS-PAGE and Western blotting, as described above.

### MMP2 activity measurement

To measure MMP2 activity, fluorogenic MMP2/MMP-9 substrate (#444215, calbiochem, CA) were utilized. Briefly, 500 μg of tissue lysate homogenized under various reducing conditions (0.1% to 5 % of SDS in homogenization buffer) were reacted with final fluorescent substrate concentration of 50 μM. The resulting mixtures were than incubated for 24 hours at 37 °C in a 96-well black plate. The fluorescence (300 nm/ 360 nm) was measured using a plate fluorescence reader (Berthold Technologies.).

### Histopathological analysis

Kidneys were fixed in 10 % neutral formalin and paraffin-embedded sections were stained with hematoxylin and eosin (H&E staining). Immunohistochemistry staining for kidney A2M was performed by using A2M antibody. Formalin-fixed, paraffin-embedded kidneys were stained by using a kit (DAB based IHC systems; Enzo, Farmingdale, NY, USA) according to manufacturer’s instructions. Kidneys were fixed in 10% neutral formalin and paraffin-embedded sections were stained with hematoxylin and eosin (H&E staining). Sirius-red staining and Masson’s Trichrome staining were done to detect the degree of fibrosis that had occurred during aging were performed as described previously [32].

### Statistical analysis

Analysis of variance (ANOVA) was used to analyze differences among all groups. Differences in the means of individual groups were assessed using the Fisher’s protected least-significant difference *post hoc* test. Student’s t-test was used to analyze differences between two groups. Values of *P* < 0.05 were considered statistically significant. Analyses were performed by using GrapdPad Prism 5 (GraphPad Software, La Jolla, CA, USA).

## SUPPLEMENTARY MATERIALS FIGURES AND TABLES


