# Supplementary material for: MMP2-A2M interaction increases ECM accumulation in aged rat kidney and its modulation by calorie restriction
Source: Oncotarget. 2017 Dec 24;9(5):5588–99. doi: 10.18632/oncotarget.23652 (PMC5814160; doi:10.18632/oncotarget.23652)
Supplement: Supplementary file 1 [file oncotarget-09-5588-s001.pdf]

MMP2-A2M interaction increases ECM accumulation in aged rat kidney and its modulation by calorie restriction

SUPPLEMENTARY MATERIALS

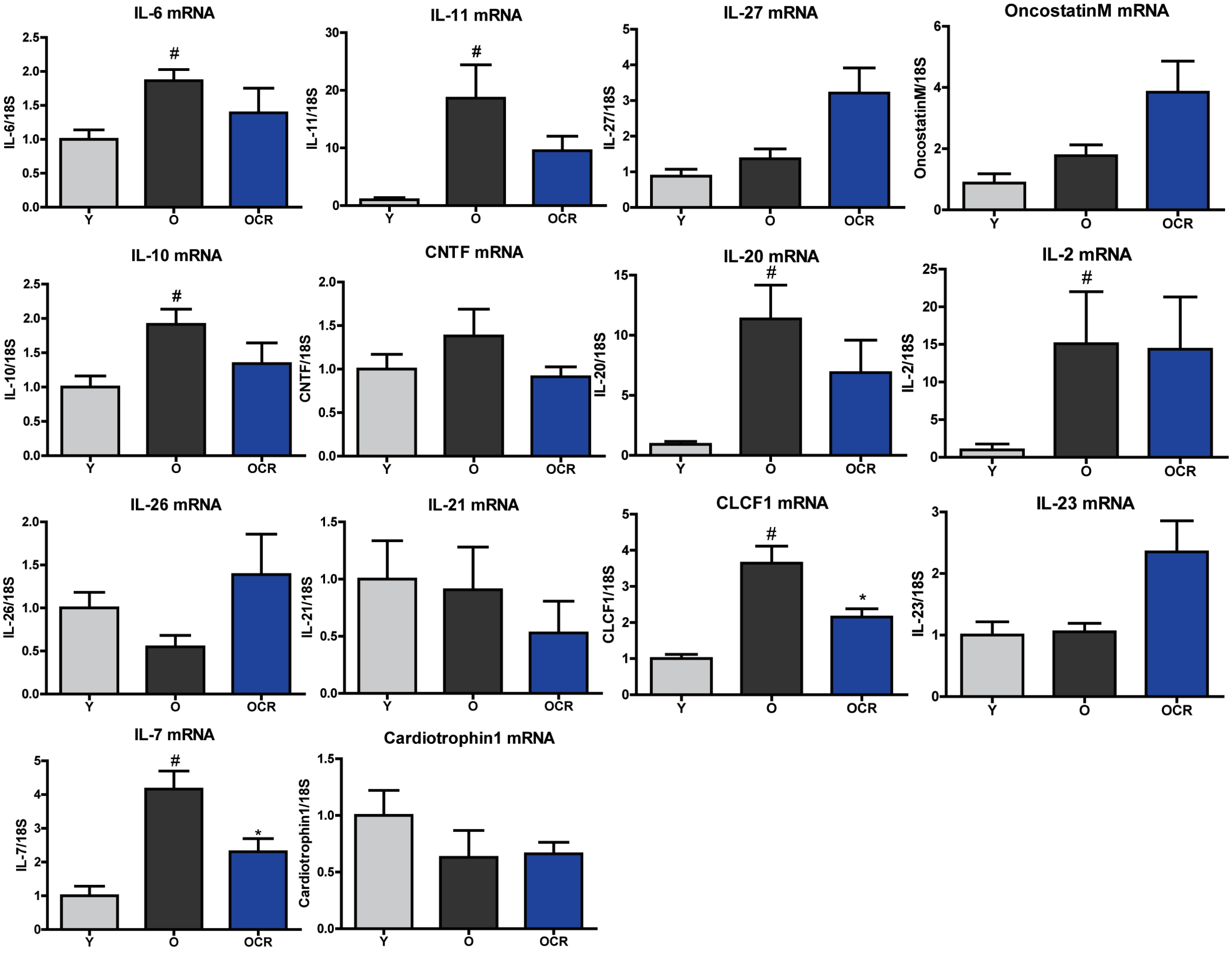

Supplementary Figure 1: Screening of STAT-3 activating cytokines in kidney

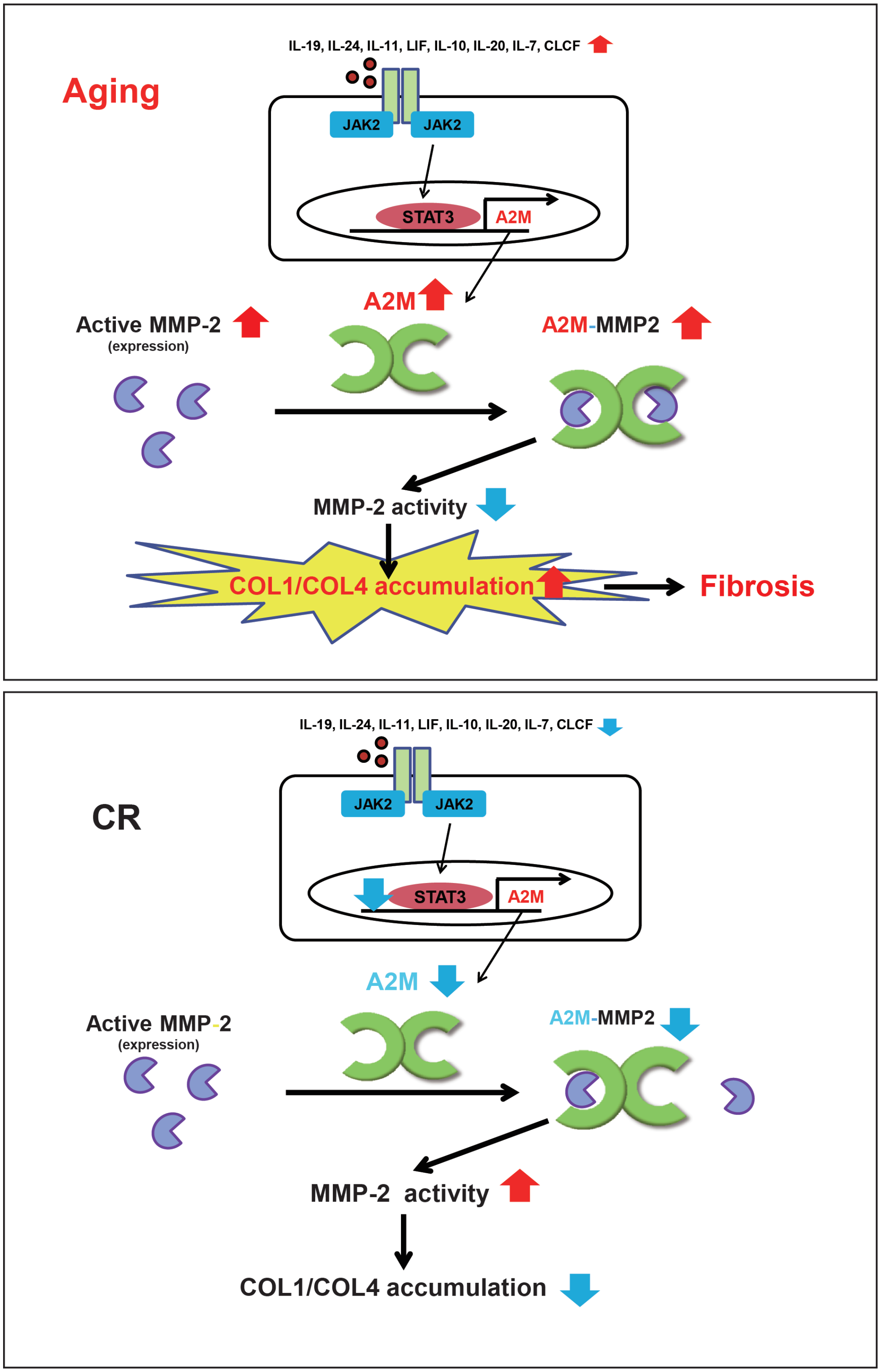

Supplementary Figure 2: Graphical abstract

Supplementary Table 1: Primary antibodies used in experiments

Antibody	Host animal	Dilution	Company
MMP-2	Mouse	1:1000	Santa Cruz (sc-13594)
COL1	Mouse	1:1000	Santa Cruz (sc-80760)
COL4A1/3	Goat	1:1000	Santa Cruz (sc-9301)
A2M	Rabbit	1:1000	Proteintech (13545-1-AP)
β-actin	Mouse	1:2000	Santa Cruz (sc-47778)
TF-IIB	Rabbit	1:1000	Santa Cruz (sc-225)
p-STAT3 (Tyr705)	Rabbit	1:1000	Cell Signaling (#9131)

Supplementary Table 2: Primer sequences for qPCR

**Gene**	**Forward (5'-3')**	**Reverse (3'-5')**
MMP2	TGT CTC CCC CAA AAC AGA CA	CAT CTG GGT TGC CAC ATC TT
A2M	AAA GTC TGC GAA CGG CTC AG	CAC TTC AGC CAC TCC TGC TG
IL-6	TCA TTC TGT CTC GAG CCC AC	GAA GTA GGG AAG GCA GTG GC
IL-11	TAG CAA ACG GAC CGA AAC AC	TGC GTG ACT TT GCT GAA AC
LIF	AAA AAC CTC AAC CCC ACT GC	TTG TTG CAC AGA CGG CAA AG
OncostatinM	TCC TGT TCC TGA GCA TGT CAT G	AGC GTG TTC AGG TTT TGG TG
CNTF	ACA GAA GCA AAC CAG CTC AC	AGG TGT TTG CTC TGC GAA AG
IL-27	TTT GCT GAA TCT CGC TTG CC	TCA GGG AAA CAT TGG GGA GAT G
IL-10	AAG ACC AGC AAA GG CAT TC	TAT GTT GTC CAG CTG GTC CTT C
IL-19	ACA GCT GCC TTC CAT CGT TG	GAG GCG CAT GTC CAC AGA AA
IL-20	TTG TGG CGA AGA AGC AAT GG	AGA ATG CCT AGT TCC CCC AAA G
IL-22	TCA ACC GCA CCT TTA TGC TG	TCA AGG GTG AAG TTG AG AC
IL-24	GGA CAA TGC CAT GCT TCC CA	GGC ACGGAG AA AAA GGA GG
IL-26	TTG CCA TTG CCA AGC ACA AG	ATC GTT GCT TTG AGC CAT GC
IL-2	TGC AGC GTG TGT TGG ATT TG	TGG CTC ATC ATC GAA TTG GC
IL-7	AAT TCC TCC CCT GAT CCT TGT G	CAA AGG CTT TAC CGT CCT TGT C
Cardiotophin1	ACA GAC ACA CAA CCT TGC AC	GTT GCT GCA CAT ATT CCT CCA G
CLCF1	TGC TTT CTG CTT GCC ACT TG	AAA GGC ACA TGC TAC AGT GGF
IL-21	CCA TAA ATC AAG CCC CCA AAG G	TGC TCA CAT TGC CCC TTT AC
IL-23	CAG TGT GGT GAT GGT TGT GAT C	TTG GCG GAT CCT TTG CAA AC
GAPDH	TGC TGG TGC TGA GTA TGT CG	AGT TGG TGG TGC AGG ATG C
18S	GAC AAC AAG CTG CGT GAG GA	GTG GTC TTG GTG TGC TGA CC
